# Time of onset of hematological side effects with Clozapine

**DOI:** 10.1192/j.eurpsy.2022.1876

**Published:** 2022-09-01

**Authors:** H. Ktari, A. Ouertani, S. Madouri, A. Aissa, Y. Zgueb, U. Ouali, R. Jomli

**Affiliations:** 1 Razi hospital, Psychiatry A Department, Manouba, Tunisia; 2 Razi hospital, Psychiatry A Department, manouba, Tunisia

## Abstract

**Introduction:**

Clozapine use is not deprived of serious complications that can condition treatment strategies, particularly hematological. Recognizing the time it takes for these effects to set, can therefore help to better screen their appearance, improving healthcare.

**Objectives:**

To study the time of onset of hematological adverse reactions in patients treated with Clozapine.

**Methods:**

A longitudinal, retrospective and descriptive study on a period of 20 years starting from the first of January 2000, at the psychiatry department A of the Razi hospital in Tunisia. This study was conducted on patients treated by Clozapine. The data was collected from patients’ medical files using a pre-established sheet.

**Results:**

The studied sample included 64 patient. Hematological disorders were found in 21 patients (32.8%). The mean time of onset of hematological adverse reactions was 119.71±126.56 days. Indeed, some patients had presented more than one hematological disorder and this at different times. Mild to moderate neutropenia had a mean time of onset of 502.57±908.32 days. The time of onset of eosinophilia was 937.75±1725.87 days, 297.67 ± 444.93 days for thrombocytopenia, 741±1268.85 days for leukopenia, 69.25 ± 48.19 days for hyperleukocytosis and 183. 33±231.80 days for anemia. Two cases of agranulocytosis were noted: one case occurred 10 years and three months from treatment beginning and the second case occurred after 7 months of treatment onset.

**Conclusions:**

The time of onset of hematological side effects with clozapine varies widely and cannot be predicted with precision. Early, more frequent and regular surveillance is therefore necessary in this population.

**Disclosure:**

No significant relationships.

